# Proteasome dynamics in response to metabolic changes

**DOI:** 10.3389/fcell.2025.1523382

**Published:** 2025-03-03

**Authors:** Cordula Enenkel, Oliver P. Ernst

**Affiliations:** ^1^ Department of Biochemistry, University of Toronto, Toronto, ON, Canada; ^2^ Department of Molecular Genetics, University of Toronto, Toronto, ON, Canada

**Keywords:** metabolic regulation of proteasome localization, proteasome condensates in membraneless organelles, proteasome storage granules, protein homeostasis (proteostasis), ubiquitin 26S-proteasome system

## Abstract

Proteasomes, essential protease complexes in protein homeostasis, adapt to metabolic changes through intracellular movements. As the executive arm of the ubiquitin-proteasome system, they selectively degrade poly-ubiquitinated proteins in an ATP-dependent process. The primary proteasome configuration involved in this degradation is the 26S proteasome, which is composed of a proteolytically active core particle flanked by two regulatory particles. In metabolically active cells, such as proliferating yeast and mammalian cancer cells, 26S proteasomes are predominantly nuclear and actively engaged in protein degradation. However, during nutrient deprivation or stress-induced quiescence, proteasome localization changes. In quiescent yeast, proteasomes initially accumulate at the nuclear envelope. During prolonged quiescence with decreased ATP levels, proteasomes exit the nucleus and are sequestered into cytoplasmic membraneless organelles, so-called proteasome storage granules (PSGs). In mammalian cells, starvation and stress trigger formation of membraneless organelles containing proteasomes and poly-ubiquitinated substrates. The proteasome condensates are motile, reversible, and contribute to stress resistance and improved fitness during aging. Proteasome condensation may involve liquid-liquid phase separation, a mechanism underlying the assembly of membraneless organelles.

## Proteasomal protein breakdown is ubiquitin- and ATP-dependent

Protein homeostasis describes the equilibrium between protein synthesis and degradation, and involves dynamic assembly and disassembly of proteins, and their trafficking between cellular compartments (Wolf and Menssen, 2018). Newly synthesized proteins can be misfolded, be supernumerary, or missing their native interaction partner due to heterologous expression. If these proteins expose hydrophobic regions prone to random aggregation, circuits of protein quality control make triage decisions. The question arises: Should these proteins be refolded by chaperones, eliminated by degradation, or deposited into organelles? Stress complicates triage decisions. To cope with stress, chaperones are activated to preserve proteins from being degraded. Up to the early 1980s, it was not plausible that peptide bonds, which require large amounts of energy to be built, could be reverted. Only the discovery of ubiquitin-mediated protein degradation triggered a paradigm shift that peptide bonds are broken under ATP consumption, which was awarded with the Nobel Prize in 2004 (Giles, 2004).

In the lab environment, yeast and mammalian cancer cells are easily cultured and have plenty of energy. ATP-dependent proteolysis was recognized as an advantage to eliminate unwanted short-lived proteins. By this, biological activities of proteins, i.e., regulating cell cycle progression and gene expression, are irreversibly switched off (Goldberg, 2003). Their shutdown is achieved by protein degradation through proteasomes, the key proteases of the ubiquitin-proteasome system (UPS) (Hershko and Ciechanover, 1998). Ubiquitin serves as a death signal and is conjugated in multiple copies to protein substrates for recognition by the proteasome. The ubiquitin moieties are linked to the substrate through reiterating cycles of ATP-consuming ubiquitin activation and ligation (Ciechanover, 2015). Thus, poly-ubiquitination of protein substrates is highly ATP demanding. The unfolding and translocation of protein substrates into the proteolytic cavity of proteasomes further consume hundreds of ATP molecules (Benaroudj et al., 2003; Peth et al., 2013). Ubiquitination is also involved in the elimination of proteins by the vacuole/lysosome, which engulfs cytoplasmic constituents and cell surface receptors via autophagic and endosomal vesicles (Ciechanover, 2005). Lysosomal degradation targets long-lived proteins, membrane-associated proteins, protein aggregates, and macromolecular machineries such as proteasomes (Ballabio and Bonifacino, 2020; Hoeller and Dikic, 2016; Marshall and Vierstra, 2019).

## Proteasome structure

**FIGURE 1:**
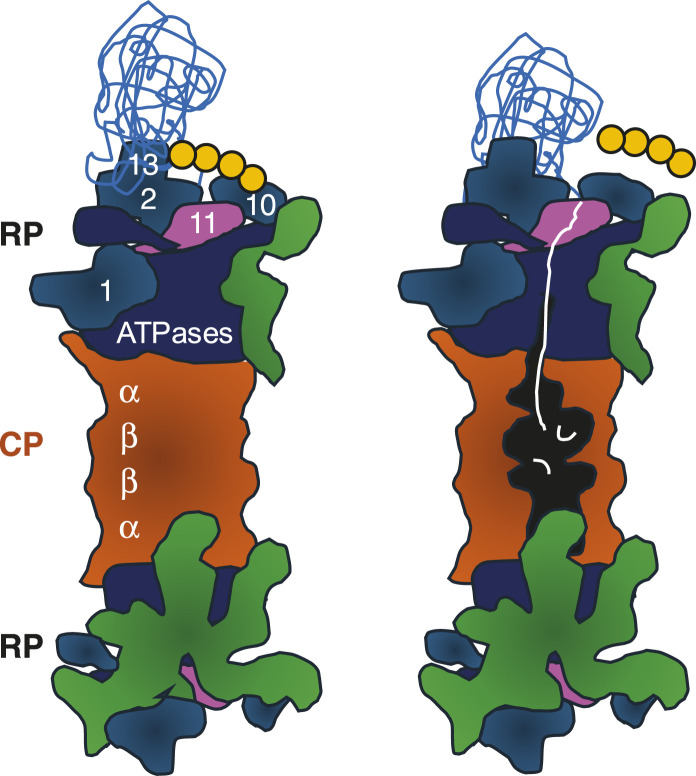
Cartoon of 26S proteasomes with RP-CP-RP configuration. Left: RP base ATPases (marine blue) and RP base subunits Rpn1, Rpn2, Rpn10, and Rpn13 recognizing the poly-ubiquitinated protein substrate (sky blue) are depicted. Four ubiquitin molecules represent the minimal poly-ubiquitin chain (yellow). RP lid subunits are depicted in green. Right: Once the poly-ubiquitin chain is cleaved off by Rpn11 (pink), the ATPases are committed to translocate the unfolded protein (white) through the outer α-rings of the CP. The unfolded polypeptide is degraded into peptides in the catalytic cavity located between the inner β-rings of the CP (orange). CP, core particle; RP, regulatory particle. The model is adopted from Matyskiela et al. (2013).

Proteasome biogenesis requires huge amounts of energy, as the proteasome is the second most abundant protein complex composed of ∼33 different subunits. Thus, proteasome biogenesis only takes place in proliferating cells with high metabolic activity (Marguerat et al., 2012). Subunit incorporation into the proteasome complex requires transient interactions (Gu and Enenkel, 2014). Their concerted action yields the proteolytic core particle (CP) with two adjacent regulatory particles (RP), known as RP-CP-RP configured 26S proteasome (Figure 1) (Hershko and Ciechanover, 1998; Tanaka, 2009). Asymmetric RP-CP configurations also exist under the name of 26S proteasomes but are not further dealt with in this review.

The RP is divided into a base and lid subcomplex (Glickman et al., 1998). The RP base contains, among other subunits, Rpn1, Rpn10 and Rpn13, both bridged by Rpn2, which recognizes the poly-ubiquitin chain of the substrate. In addition, several ubiquitin receptors exist, such as RAD23 in mammals and Rad23 in yeast. They transiently interact with the RP to hand over poly-ubiquitinated substrates for degradation (Shi et al., 2016). Proteasomes do not care about the nature of the substrates, just the presence of the ubiquitin death signal.

The RP base also contains a six-membered ATPase ring, which is responsible for opening/gating of the CP, substrate unfolding, and translocation. Since branched poly-ubiquitin chains are bulky, they are removed from the protein substrate prior to degradation. The isopeptide bond between the ubiquitin chain and the substrate is cleaved by the deubiquitinase Rpn8-Rpn11 module located in the RP lid (Wehmer et al., 2017; Bard et al., 2019). The release of the poly-ubiquitin chain induces a conformational switch by which the RP base ATPase ring snaps into place on the adjacent CP gate (Bard et al., 2019).

Over the last years, single-particle cryo-electron microscopy enabled the deconvolution of coexisting 26S proteasome conformations and their delineation in the degradation of poly-ubiquitinated substrates (Wehmer et al., 2017; Unverdorben et al., 2014; Schweitzer et al., 2016). The recognition of the poly-ubiquitinated substrate occurs in the inactive s1 ground state of the 26S proteasome. The 26S proteasome then adopts several commitment states until substrate degradation becomes irreversible (Wehmer et al., 2017; Unverdorben et al., 2014; Dong et al., 2019; de la Pena et al., 2018; Eisele et al., 2018). These proteasome rearrangements resulting in conformational heterogeneity prevented crystallographic analyses of the 26S proteasome and RP. It is worth mentioning that 26S proteasomes are further able to cleave proteins with intrinsically disordered regions in an ubiquitin-independent manner (Erales and Coffino, 2014), sometimes leading to protein processing through limited proteolysis (Rape and Jentsch, 2002). Sophisticated *in vitro* experiments revealed that a folded protein is spared from degradation although being modified by a poly-ubiquitin chain. Instead, an intrinsically disordered protein lacking poly-ubiquitination but interacting with the folded poly-ubiquitinated protein was degraded (Inobe and Matouschek, 2014). On one hand, this suggests that poly-ubiquitination is not necessarily leading to proteasomal degradation (Collins and Goldberg, 2017). On the other hand, proteins with intrinsically disordered regions are sensitive to proteasomal degradation and can have shorter half-life (Tsvetkov et al., 2009; van der Lee et al., 2014).

Furthermore, 26S proteasome assembly is sensitive to oxidative stress. Oxidative stress, e.g., induced by perhydrol, results in the dissociation of 26S proteasomes into the CP and RP, and an accumulation of poly-ubiquitinated substrates. Under these conditions, an increased association of the proteasome-interacting protein Ecm29 with purified RP was detected (Wang et al., 2010), consistent with the finding that Ecm29 fulfills quality control functions in proteasome assembly (Lehmann et al., 2010; Park et al., 2011). The resulting free CP has closed gates and thus latent enzyme activity (Eytan et al., 1989). However, the CP gates are accessible for intrinsically disordered and oxidatively damaged proteins that are *in vitro* degraded by the CP (Tsvetkov et al., 2009; Liu et al., 2003; Ben-Nissan and Sharon, 2014).

In contrast to the 26S holoenzyme, the CP with its more static global structure is resolved at atomic resolution by x-ray crystallography. The CP is composed of a stack of two inner β-subunit rings and two outer α-subunit rings. The inner β-rings harbor the active sites for endoproteolytic peptide bond cleavage. Outer α-rings serve as gates into the CP cavity, which are opened by the adjacent ATPase rings of the RP base. Thus, ATPase activity is required for α-ring opening, unfolding, and translocating of substrates into the CP (Groll et al., 1997).

## Nuclear proteasome localization in dividing cells

On top of conformational plasticity, 26S proteasomes are highly dynamic regarding their intracellular localization (Enenkel, 2014a; Tomita et al., 2019; de Almeida et al., 2021). Our understanding of proteasome localization in cells was debated for decades before a consensus was reached.

In mammalian cells, intracellular proteasome localizations by indirect immunofluorescence microscopy had been controversially discussed as they varied depending on antibodies, cell lines, and culture conditions used (Brooks et al., 2000). At high confluency, when nutrients became limiting in the cell culture medium, proteasomes appeared to be cytoplasmic, while proteasomes appeared to be more nuclear in cancer cells grown at low confluency (Wojcik and DeMartino, 2003). Early indirect immunofluorescence microscopy using antibodies with cross-reactivity for proteasomes from different organisms revealed intracellular distributions of proteasomes. Proteasomes were localized to the nucleus in *Xenopus laevis* oocytes and HeLa cells (Peters et al., 1994). Particularly in the prophase of rat granulosa cells, proteasomes accumulated with chromatin, where also cyclins localize before being degraded (Amsterdam et al., 1993). Cyclins are short-lived proteins regulating cell cycle progression and one of the first identified proteasomal substrates (Glotzer et al., 1991). At that time, the detection of nuclear proteasomes was consistent with Varshavsky’s and co-workers’ discovery that ubiquitin-dependent protein degradation plays a critical role in cell cycle control and gene expression (Finley et al., 1984). Four decades later, proteasome abundance in the nucleus is still attracting attention, with quantification by nuclear fractionations and proteomics analyses confirming cell cycle-dependent recruitment of proteasomes to chromatin (Kito et al., 2020). Meanwhile, monoclonal antibodies that enable the co-immunoprecipitation of 26S proteasomes are commercially available and suitable for proteasome localization by indirect immunofluorescence microscopy (Hendil et al., 1995). Complementary to this classical approach, the labeling of proteins with green fluorescent protein (GFP) and related variants became an invaluable technique to correlate cell cycle-dependent dynamics of protein concentrations and their localizations using live-cell imaging (Litsios et al., 2024). In yeast, the chromosomal replacement of proteasomal subunits by GFP-labeled versions is standardized and yields reliable fluorescent reporter subunits that are fully incorporated into proteasomes. Almost every proteasomal subunit is functionally replaceable by a GFP-labeled version consistently showing the same intracellular distribution in yeast (Enenkel, 2014a). In mammalian cells, an increasing number of GFP reporter subunits for live-cell imaging of proteasomes is emerging. Dantuma and co-workers were one of the first who aimed for the stable expression of GFP-labeled CP subunit α4 in cancer cell lines (Gierisch et al., 2020). The efficiency of the reporter subunit incorporation into proteasomes was verified by glycerol gradient ultracentrifugation, which separates 26S proteasomes in fast-migrating fractions from not fully incorporated subunits in slow-migrating fractions (Salomons et al., 2010). Direct fluorescence microscopy of GFP-labeled α4 in Mel JuSo cells revealed significant nuclear localization (Enenkel, 2014a). We adopted this expression system to U2OS cells and confirmed major nuclear proteasome localization for GFP-labeled α4, consistent with indirect immunofluorescence microscopy using commercial MCP444 antibodies (unpublished results). Similar observations were reported by Murata and co-workers, who established RP lid subunit Rpn11-Flag-EGFP tag-exchangeable knock-in mice. Their approach allows one to distinguish between young, in other words, newly synthesized, proteasomes in the nucleus and old proteasomes in the cytoplasm of embryonic fibroblasts. Thus, this cell system is suited to monitor age-related proteasome dynamics in mammalian cells (Tomita et al., 2019). More recently, Zuber and co-workers developed an elegant approach by ectopic expression of fluorescent mCherry-labeled proteasomal subunits in CRISPR-Cas9 induced RKO knockdown cells. Their approach yielded the full replacement of the endogenous CP β4 subunit by a fluorescent-labeled version. Again, the fluorescent reporter subunit of the proteasome revealed nuclear localization in dividing RKO cells (de Almeida et al., 2021).

Taken together, direct and indirect fluorescence microscopy in proliferating yeast and mammalian cells with high metabolic activity reveal nuclear localization of proteasomes. It is not surprising that the localization of an essential and abundant protease complex is evolutionarily conserved (Botstein and Fink, 2011). However, even in yeast as model organism of eukaryotic cells, it was puzzling that proteasomes were primarily nuclear (Russell et al., 1999; Enenkel et al., 1998; Wilkinson et al., 1998). At this point, we would like to point out again that proteasomes in the cytoplasm are proteolytically active. Cryo-electron tomography (cryo-ET), a non-invasive imaging technology that preserves protein structures in their native cellular environment (Baumeister, 2022), of cytoplasmic volumes of neuronal cells revealed that ∼20% of the cytoplasmic proteasomes were engaged in substrate degradation. The remainder of 26S proteasomes was in the substrate-accepting ground state (Asano et al., 2015). Without stress, the reservoir of cytoplasmic 26S proteasomes appears to be far from exhausted.

## Nuclear import of proteasomes

The answer to the question of how proteasomes are imported into the nucleus is that several pathways are used. Our previous reviews have recapitulated in detail the discoveries on nuclear import of proteasomes over the last decades (Enenkel, 2014b; Wendler and Enenkel, 2019; Enenkel et al., 2022). We briefly summarize the basic concepts of nuclear import of proteasomes. In proliferating yeast, inactive CP precursor complexes and RP subcomplexes are imported by the conventional import receptor importin/karyopherin αβ, suggesting that holoenzymes are assembled in the nucleus (Lehmann et al., 2002; Isono et al., 2007). Alternatively, proteasomes are imported as matured enzymes by importins/karyopherins with transient accessory proteins such as Sts1 binding to the RP lid in yeast (Chen et al., 2011; Budenholzer et al., 2020), and AKIRIN2 binding to the CP α-ring in mammalian cells (de Almeida et al., 2021). Intriguingly, Sts1 and AKIRIN2 are short-lived. Their proteasomal degradation is triggered upon arrival in the nucleus by a mechanism not fully understood.

When yeast cells rest in quiescence, a temporary halt of proliferation, CP precursor complexes are unavailable due to stalled proteasome biogenesis. Upon exit from quiescence, nuclear proteasome assembly from newly synthesized precursor complexes takes time (Laporte et al., 2008). Thus, matured proteasomes are immediately transported from the cytoplasm into the nucleus. Sudden changes in metabolic activities, i.e., from low state in quiescence to high state in proliferation, require quick adaptations. With the resumption of cell proliferation, Blm10 facilitates nuclear import of the CP in yeast (Weberruss et al., 2013). PA200, the mammalian counterpart of Blm10, is similarly involved in nuclear proteasome activation (Ustrell et al., 2002). Which nuclear import pathway prevails over another depends on the availability of proteasomal transport cargoes, importins/karyopherins, and adaptor proteins, showcasing the plasticity of proteasome configurations under different growth conditions. To put it simply, all nuclear import pathways have in common that 26S proteasomes do not pass the nuclear pore as active enzymes. The fact that 26S proteasomes and free CP are able to degrade intrinsically disordered proteins would make the passage of active enzymes detrimental to nuclear pore proteins, because nuclear pore proteins with repetitive hydrophobic Gly-Leu-Phe-Gly motifs are intrinsically disordered (Denning et al., 2003; Denning and Rexach, 2007). To avoid collateral damage to nuclear pore proteins, proteasomes are translocated as inactive enzymes. On the way through the nuclear pore, proteasome activity is inhibited either as a precursor complex or by binding to accessory proteins, such as Blm10, which seals the CP gate.

## Proteasome condensates in response to stress and metabolic challenges

In the 2000s, the UPS field predominantly focused on cytoplasmic protein degradation by proteasomes because the scientific community was interested in endoplasmic reticulum (ER)-associated protein degradation, antigen processing, and the removal of newly synthesized proteins (Sommer and Wolf, 1997; Kloetzel and Ossendorp, 2004; Yewdell, 2005). Experiments were designed to study newly synthesized proteins that were often more expressed than their binding partners. However, Hartl and colleagues found that endogenous nascent polypeptides remain largely protected from proteolysis due to the abundance of cytoplasmic chaperones (Vabulas and Hartl, 2005). Moreover, misfolded proteins were found to be delivered into the nucleus for proteasomal degradation (Park et al., 2013), while tumor suppressor protein p53 was proposed to be exported into the cytoplasm for proteasomal degradation (Hirayama et al., 2018). The fate of p53 is intriguing because it is controlled by mono- or poly-ubiquitination, the latter has been shown to promote degradation in the nucleus (Li et al., 2003). 26S proteasomes are also engaged in cytoplasmic protein breakdown. Since 26S proteasomes are enzymes, quality counts over quantity. Sites of proteasome localizations may not correlate with major sites of proteolysis. Furthermore, the activities of nucleo- and cytoplasmic 26S proteasomes are differently regulated by post-translational modifications (Sha et al., 2011; VerPlank and Goldberg, 2017). Therefore, based on our current knowledge, it is difficult to decide in which compartment proteasomes are most active in protein degradation.

## Proteasome condensates in the nucleus of mammalian cells

Based on indirect immunofluorescence localization studies, von Mikecz and co-workers shifted the research focus back to the UPS within nuclear speckles. Intrigued by the observation that ubiquitin, the ubiquitin-activating enzyme, ubiquitin ligases, and proteasomes accumulate in nuclear speckles, also known as foci, bodies, and granules, the question arose: What is the function of these UPS conglomerations (von Mikecz, 2006)? When all UPS players are in place, it is conceivable that short-lived proteins that regulate cell cycle progression and gene expression, such as cyclins and transcription factors, are instantaneously poly-ubiquitinated and degraded on site. If so, these conglomerations represent enhanced UPS activities and differ from pathological aggregates that accumulate undegradable proteins linked to neurodegenerative disorders and can be caused by nanoparticles (von Mikecz et al., 2008).

Stress-adaptable Promyelocytic leukemia protein (PML)-associated nuclear bodies (PML NB) or clastosomes (Lafarga et al., 2002; Lallemand-Breitenbach et al., 2001) have multifaceted roles by recruiting a variety of unrelated proteins in response to stress, i.e., oxidation. PML NB are archetypes of membraneless organelles with a diameter of ∼1 µm (Sahin et al., 2014). Membraneless organelles are fascinating subcompartments, as they are thought to float as dense phase in the dilute phase of an aqueous environment. Their components often condense in response to cellular stress and dilute upon stress relief (van Leeuwen and Rabouille, 2019).

The current model to describe the phenomenon of condensed mixtures of macromolecules is liquid-liquid phase separation (LLPS). LLPS is driven by concentration gradients, the promiscuity and multivalency of macromolecules, i.e., variable weak interactions through proteins with repetitive sequences of hydrophobic amino acids, low complexity or intrinsically disordered regions, and proteins in folding transitions (Alberti and Hyman, 2021; Vernon and Forman-Kay, 2019).

PML has an intrinsically disordered C-terminal domain and interacts with multiple proteins. According to immunogold electron microscopy and confocal microscopy, PML surrounds PML NB (Silonov et al., 2023; Lallemand-Breitenbach and Hugues, 2010). Like a sponge, PML NB serves as overflow compartment for nuclear quality control and hosts misfolded proteins under conditions of proteotoxic stress, such as proteasome inhibition. Defective ribosomal products (DRiPs), in other words, aberrant newly synthesized proteins of low molecular mass, constantly escape the cytoplasmic quality control system. They diffuse through nuclear pores into the nucleus, are ubiquitinated, and transiently stored in PML NB (Silonov et al., 2023; Lallemand-Breitenbach and Hugues, 2010). The condensed PML NB core could be envisioned as a unique solvent continuously extracting and exchanging proteins from the environment. Heat shock proteins, chaperones, and proteasomes around PML NB reduce the influx of DRiPs by refolding and degradation, respectively. Thus, PML NB have a highly dynamic DRiP composition, and prevent unintended interactions of DRiPs with nuclear proteins. Failures of DRiP clearance under conditions of prolonged stress, such as critical energy shortage and irreversible proteasome inhibition, result in PML NB solidification. Immobilization of UPS components in solidifying PML NB leads to depletion of ubiquitin and proteasomes, which jeopardizes cell vitality (Mediani et al., 2019a; Mediani et al., 2019b). The age-related and thus irreversible decline of proteasome activities (Chondrogianni et al., 2003; Torres et al., 2006) causes challenges in senescent cells that cope with the burden of poly-ubiquitinated proteins by uptake into nuclear proteasome bodies (senescence-associated nuclear proteasome foci; SANPs) using the ubiquitin receptor RAD23B (Iriki et al., 2023).

Meanwhile, evidence has increased that membraneless organelles containing proteasomes and a diversity of poly-ubiquitinated substrates originate from various stress conditions. The simplest explanation for this phenomenon is that different kinds of stress cause an energy crisis and force the energy-consuming UPS into quality control compartments. As mentioned above, stress-adaptable PML NB and SANP might represent overflow compartments for UPS clearance (Mediani et al., 2019a; Mediani et al., 2019b; Iriki et al., 2023). Additional stressful situations leading to UPS condensation are listed in Table 1. For example hyperosmotic shock induces the formation of nuclear organelles containing proteasomes, poly-ubiquitinated proteins, chaperone VCP p97, and ubiquitin receptor RAD23B. Orphan ribosomal subunits that failed to be incorporated into nascent ribosomes represent an abundant source of proteasomal substrates and are part of these organelles. These organelles were one of the first to be resolved by cryo-ET analysis. 26S Proteasomes were found to be randomly distributed within the organelle. *In vitro,* LLPS was mediated by multivalent interactions between two ubiquitin-associated domains of RAD23B and tetraubiquitin chains, two components of this UPS organelle (Yasuda et al., 2020).

**TABLE 1:** Proteasome organelles induced by different stress factors.

Name	Stress factor	Organism	Locali-zation	Composition	Methods	Disassembly	Ref
nUPS speckles	nanoparticles, UPS inhibition	M	N	UPS, poly-Ub proteins	IF	Proteasomes	von Mikecz (2006), von Mikecz et al. (2008)
PML NB	inflammation, viral infection, oxidation, proteasome inhibition	M	N	PML, misfolded proteins, DRiPs, ubiquitination, SUMOylation, surrounded by UPS	IF, IEM	Proteasomes	Lafarga et al. (2002), Lallemand-Breitenbach et al. (2001), Sahin et al. (2014), Silonov et al. (2023), Lallemand-Breitenbach and Hugues (2010), Mediani et al. (2019a), Mediani et al. (2019b)
SANP	senescence	M	N	proteasome, poly-Ub proteins, RAD23B	IF, foci fusion, FRAP	n. d.	Iriki et al. (2023)
INQ	proteasome inhibition, DNA damage	Y	NE -N	proteasome shell, poly-Ub, misfolded proteins, SUMOylation	IEM, FM, FLIP, foci fusion	proteasomes, chaperones	Miller et al. (2015), Kumar et al. (2022)
JUNQ, aggresome	proteasome inhibition, substrate overexpression	Y, M	NE - ER	proteasome shell, poly-Ub, misfolded proteins	IF, FLIP, FRAP, FM	proteasomes, chaperones	Kaganovich et al. (2008), Kopito et al. (2000)
proteasome foci	high glucose, hyperosmotic shock	M	N	proteasome shell, poly-Ub proteins, orphan ribosomal proteins, RAD23B, VCP p97 chaperone	TEM, FM, cryo-ET, FRAP, LLPS	isoosmosis, DUB	Yasuda et al. (2020)
SIPAN	amino acid starvation	M	N	proteasome, poly-Ub proteins, RAD23B	IEM, FM, FRAP, LLPS	amino acids, DUB	Uriarte et al. (2021)
p62 foci	heat, oxidation, inhibited nuclear export by Crm1/Xpo1	M	N	core: p62, poly-Ub proteins, orphan proteasomal subunits; shell: proteasome, ubiquitination cascade	FRAP, FM, foci fusion	stress relief	Fu et al. (2021)
GA aggregates	neurotoxic Gly-Ala repeats	M	C	proteasomes stalled in degradation of neurotoxic proteins occurring in amyotrophic lateral sclerosis and frontotemporal dementia	cryo-ET	irreversible	Guo et al. (2018)
PSG	glucose starvation, low ATP, low pH	Y	C	proteasome, monoubiquitin	IEM, FM	glucose, high ATP	Laporte et al. (2008), Gu et al. (2017), Waite et al. (2022), Peters et al. (2013), Saunier et al. (2013), van Deventer et al. (2015)

Abbreviations: C, cytoplasm; cryo-ET, cryo-electron tomography; DRiP, defective ribosomal product; DUB, deubiquitinase; FLIP, fluorescence loss in photobleaching; FM, direct fluorescence microscopy; FRAP, fluorescence recovery after photobleaching; IEM, immunoelectron microscopy; IF, indirect immunofluorescence microscopy; LLPS, *in vitro* reconstitution of liquid-liquid phase separation; n. d., not defined; NE, nuclear envelope; N, nucleus; M, mammals; TEM, transmission electron microscopy; Ub, ubiquitin (ated); Y, yeast.

Heat, oxidation, and inhibition of nuclear export through the canonical export receptor Crm1/Xpo1 are alternative stressors. They trigger nuclear LLPS of poly-ubiquitinated proteins, including orphan proteasomal subunits that escaped the incorporation into precursor complexes in the cytoplasm. Notably, the receptor p62 for transport of ubiquitinated cargo into autophagosomes is sequestered into these nuclear foci. Confocal immunofluorescence localization studies revealed that proteasomes and enzymes of the ubiquitination cascade are at the periphery of these foci, suggesting a local enhancement of UPS activities (Fu et al., 2021).

Acute amino acid deprivation is another stressor. It triggers the reversible formation of starvation-induced proteasome assemblies in the nucleus (SIPAN) with poly-ubiquitinated proteins shuffled by RAD23B. RAD23B is highly intrinsically disordered and undergoes LLPS in the presence of crowding agents, i.e., Ficoll, dextran or polyethylene glycol (Uriarte et al., 2021). *In vivo*, SIPAN is dissolved by amino acid replenishment and contributes to stress resilience and fitness under pathological conditions (Uriarte et al., 2021). Following the starvation of amino acids, specifically of the mTOR-agonistic aromatic amino acids Phe, Tyr, and Trp, the proteasome moves from its large nuclear pool to the cytoplasm (Livneh et al., 2023). This phenomenon mirrors early observations of proteasome movements from the nucleus to the cytoplasm in response to increased confluency of mammalian cells in culture (Wojcik and DeMartino, 2003).

## Proteasome condensates in the nuclear periphery of mammalian cells

At both the nucleo- and cytoplasmic side of the nuclear envelope (NE), the latter connected with the ER, proteasomes and substrates were found to be condensed in intranuclear quality (INQ) and juxta-nuclear quality (JUNQ/CytoQ) speckles, respectively. These cellular ‘junkyards’ were initially observed when proteasomal degradation was overwhelmed through heterologous expression of model substrates, e.g., the von Hippel Lindau protein in yeast (Kaganovich et al., 2008; Kopito, 2000; Miller et al., 2015). JUNQ formation is fostered by proteasome inhibition either chemically in mammalian cells or by UPS-specific mutations in yeast. When stress relieves, e.g., by proteasome and chaperone activation, the ‘junkyards’ dissolve (Kaganovich et al., 2008). Interestingly, INQ and PML NB are discussed to represent counterparts in yeast and humans, respectively, and depend on SUMO-ylation, an ubiquitin-like modifier, which distinguishes INQ and PML NB from JUNQ (Kumar et al., 2022). Failures in the clearance of these quality control compartments are thought to be related to neurodegenerative disorders and premature aging (Kopito, 2000; Miller et al., 2015).

To understand the occurrence of UPS-containing organelles on either nucleo- or cytoplasmic side of the NE, the kinetics of UPS transport through nuclear pores might be considered. Cryo-ET of *Chlamydomonas* cells revealed 26S proteasomes tethered to the basket of the nuclear pore, at the inner NE (Albert et al., 2017) and in clusters at the outer NE/ER for ER-associated protein degradation (Albert et al., 2020). As the diameter of nuclear pores shrinks upon stress and energy depletion and dilates with stress relief and energy replenishment (Zimmerli et al., 2021), UPS components and poly-ubiquitinated substrates tend upon stress to be concentrated on either side of the bottleneck formed by nuclear pores. Thus, phase separation of proteasomes and poly-ubiquitinated substrates could be the result of molecular crowding due to impaired nuclear transport. In the event of extreme stress, UPS organelles may even fragment nuclear pore components, but this hypothesis remains to be tested (Lee et al., 2021).

**FIGURE 2:**
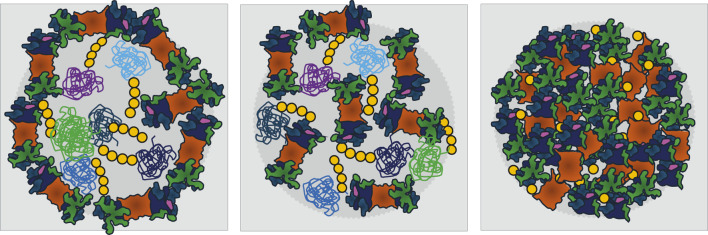
Models for membraneless organelles containing the ubiquitin-proteasome system (UPS) that form in response to nutrient limitations and stress. For simplicity, only proteasomes, the key proteases of the UPS, are depicted. Left: A shell of proteasomes surrounds a core of poly-ubiquitinated proteins. Middle: UPS components are randomly distributed between poly-ubiquitinated proteins. Both types of organelles are proposed to serve as proteolysis centers and to be driven by liquid-liquid phase separation (LLPS). Right: In quiescent yeast, proteasome storage granules (PSGs) behave differently as they contain no poly-ubiquitinated proteins. PSG formation requires the presence of mono-ubiquitin above a threshold, for details see Table 1. Proteasomes are depicted as in Figure 1. The variety of poly-ubiquitinated proteins is symbolized by green, violet and blue colors. Ubiquitin is yellow.

To gain an overview of the repeating patterns of UPS organelles that harbor proteasomes or are surrounded by a proteasome shell (Figure 2), the common theme is stress, which ultimately requires energy management. Macromolecular machineries such as proteasomes may simply throttle their motor once energy is scarce. Further intriguing questions are: How many different UPS organelles coexist? To answer this question, we will focus future research on the analysis of proteasome configurations rather than individual substrates within different organelles. And how many more energy-dependent macromolecular machineries simultaneously condense into membraneless organelles to overcome stressful conditions (Gu et al., 2017)?

## Proteasome condensates in the cytoplasm of mammalian cells

In the cytoplasm of mammalian cells, the presence of proteasome condensates is less explored. Instead, stress granules are known to be cytoplasmic organelles formed by LLPS. Stress granules contain non-translating RNA, a plethora of RNA-binding proteins and stalled preinitiation 40S ribosomes (Protter and Parker, 2016). Again, stress relief triggers stress granule clearance and the resumption of protein biogenesis. Stress granules do not contain proteasomes (Jain et al., 2016) but relieve the burden on the nuclear UPS by hosting misfolded proteins in the cytoplasm (Xu et al., 2023).

Close to the cytoplasmic side of the NE, proteasomes are concentrated in aggresomes, which are structurally and functionally overlapping with JUNQs. The formation of aggresomes, as well as of JUNQs, is induced by proteasome inhibition and overexpression of neurotoxic proteins. Aggresomes were initially proposed to serve as proteolytic centers (Wojcik and DeMartino, 2003; Kaganovich et al., 2008; Kopito, 2000). How inhibited proteasomes accelerate the proteolysis of toxic and sometimes undegradable proteins remains a conundrum.

Cryo-ET was employed to understand the molecular architecture of neurotoxic protein aggregates within intact neurons. A genetic aberration in the C9orf72 gene leading to modifications with repetitive Gly-Ala motifs is responsible for the development of amyotrophic lateral sclerosis and frontotemporal dementia. The hydrophobic patches of poly-Gly-Ala peptides produced by this genetic aberration are prone to aggregation. Cryo-ET revealed that undegradable aggregates containing poly-Gly-Ala peptides trap 26S proteasomes in a substrate-processing conformation, causing them to be stuck in a dead-end road of protein degradation and severely compromising protein homeostasis (Guo et al., 2018).

Notably, fluorescence microscopy cannot distinguish between proteolytically active proteasomes and proteasomes stalled in degradation, and thus cannot differentiate between reversible UPS organelles and irreversible UPS aggregates. This may explain why previous histograms of centenarians’ brains showing aggregations and inclusions of proteasomes and ubiquitin were difficult to interpret. UPS organelles were not necessarily associated with a medical history of neurological diseases (Tanaka and Matsuda, 2014; Ciechanover and Kwon, 2017; Itoh et al., 1998). As long as UPS organelles remain reversible, they confer fitness during aging (Iriki et al., 2023).

Can we nowadays characterize reversible UPS organelles? Unfortunately, membraneless UPS organelles fall apart during cell disintegration, unless they are chemically fixed. They escape biochemical characterization by conventional means. Cryo-ET became the state-of-the-art technology to provide insight into the structure and function of UPS organelles without interfering with their native environment (Baumeister, 2022).

## Proteasome storage granules in the cytoplasm of yeast cells

In yeast cells transitioning from logarithmic to stationary phase, cells start competing for nutrients. Due to glucose deprivation, cells become less metabolically active. Thus, the ATP concentration strikingly decreases (Laporte et al., 2011). Cell proliferation is temporarily halted, and cells enter quiescence (De Virgilio, 2012). During the transition to quiescence, yeast proteasomes uniformly move towards the NE. Proteasome clusters are detected close to nuclear pores, suggesting that proteasomes are piling up before being slowly translocated through nuclear pores (Laporte et al., 2008). During prolonged quiescence, which is marked by high cell density and low metabolic activity, yeast proteasomes eventually exit the nucleus. They then accumulate in proteasome storage granules (PSGs) in the cytoplasm. PSGs are also induced by mitochondrial malfunctions (Waite and Roelofs, 2022) and low pH due to deficient proton pumping (Peters et al., 2013), suggesting that ATP availability and further downstream metabolites of catabolic pathways influence PSG formation. Additionally, signaling cascades involving mitogen-activated protein kinase MAPK, and AMP kinase, named Snf1 in yeast, strengthen the importance of cellular energy homeostasis for PSG formation (Li et al., 2019). Acidification converts the aqueous protoplasm into a solid-like phase that restricts the mobility of macromolecules and fosters their condensation (Munder et al., 2016; Parry et al., 2014).

Upon metabolic reactivation, PSGs immediately dissolve. Within a few minutes, mature CP and RP stored in PSGs appear reassembled in the nucleus (Laporte et al., 2008; Weberruss et al., 2013). If quiescent yeast cells are disintegrated in buffer with ATP regeneration, intact 26S proteasomes are obtained. If quiescent cells are disintegrated without ATP supplement, 26S proteasomes are dissociated into RP and CP (Weberruss et al., 2013; Gu et al., 2017; Bajorek et al., 2003). Which buffer will mimic the physiological environment of PSGs? Only in the presence of a chemical cross-linker could PSGs be isolated as intact organelles (Gu et al., 2017). Mass spectrometry and biochemical analysis of cross-linked PSGs revealed a homogeneous composition of proteasomal subunits but no poly-ubiquitinated substrates, suggesting that PSGs are not active in the degradation of poly-ubiquitinated proteins. High-throughput screens using the collection of yeast null mutants suggested that proteasomal deubiquitination activities (Rpn11, Ubp6) and a threshold level of free ubiquitin promote PSG formation (Gu et al., 2017). This contrasts with the various UPS organelles containing poly-ubiquitinated substrates as mentioned above. Notably, *rpn11-m1* and *ubp6Δ* mutants do not form PSGs (Gu et al., 2017; Saunier et al., 2013). Due to the RP’s deficient deubiquitinase activity in *rpn11-m1*, the Cullin-RING E3 ligase, accounting for one-fifth of the poly-ubiquitination of proteasomal substrates, remains activated by modification through the ubiquitin-like protein NEDD8/Rub1 (Bramasole et al., 2019). Thus, the burden of poly-ubiquitinated substrates upon entry into quiescence might be incompatible with PSG formation, since the negative feedback loop of reducing the pool of poly-ubiquitinated substrates by the RP is disturbed.

The reversibility of PSGs remains during prolonged quiescence (Saunier et al., 2013; van Deventer et al., 2015). Furthermore, PSGs seem to protect proteasome assemblies from autophagy (Marshall and Vierstra, 2019; Li and Hochstrasser, 2020). The question is whether LLPS is the underlying mechanism of PSG formation. Miscellaneous protein composition is a typical feature of LLPS organelles. However, mass spectrometry of cross-linked PSGs and stochastic optical reconstruction microscopy suggested dense packing of proteasomes within PSGs (Gu et al., 2017). Will few proteasomal subunits with intrinsically disordered regions support LLPS-driven PSG formation (Aufderheide et al., 2015)? Ubiquitin is a key component of the UPS, and mono-ubiquitin is required for PSG formation. Mono-ubiquitin disrupts multivalent interactions and modulates LLPS (Dao et al., 2018). In line with this, mono-ubiquitin is essential for the disassembly of stress granules in cells recovering from stress (Franzmann and Alberti, 2021). It will be exciting to learn about the structure of PSG-related organelles in comparison with proteasome condensates containing poly-ubiquitinated proteins.

Our review started with a paradigm shift in protein homeostasis based on cellular energy homeostasis: the hydrolysis of peptide bonds of short-lived proteins is achieved by ATP-consuming ubiquitination and proteasomal proteolysis in cells with high metabolic activity. Protein homeostasis is severely impacted by metabolic imbalances that are associated with aging and diseases such as diabetes, obesity, cancer, and neurodegeneration (Ciechanover and Kwon, 2017). The master regulator of metabolic stress and proteasome activity is the TOR complex1 (Rousseau and Bertolotti, 2018). Not only adenosine nucleotides but also nicotinamide adenine dinucleotide (NAD^+^) are essential as coenzymes in a myriad of bioenergetic pathways. Cells with balanced metabolism are well prepared for energy shortages and the systemic decline of coenzymes during aging and stress, to protect the UPS from autophagy and to regulate protein degradation within UPS condensates (Marshall and Vierstra, 2019; Li et al., 2019; Karmon and Ben Aroya, 2019).
